# Distinct Requirements for Zebrafish Angiogenesis Revealed by a *VEGF-A* Morphant

**DOI:** 10.1002/1097-0061(200012)17:4<294::AID-YEA54>3.0.CO;2-5

**Published:** 2000

**Authors:** Aidas Nasevicius, Jon Larson, Stephen C. Ekker

**Affiliations:** 1 Arnold and Mabel Beckman Center for Transposon Research Department of Genetics, Cell Biology and Development University of Minnesota 6-160 Jackson Hall, 321 Church Street SE Minneapolis MN 55455 USA; 2 Biochemistry Molecular Biology and Biophysics Graduate Program University of Minnesota 6-160 Jackson Hall, 321 Church Street SE, Minneapolis MN 55455 USA

## Abstract

Angiogenesis is a fundamental vertebrate developmental process that requires signalling by the secreted protein vascular endothelial growth factor-A (*VEGF-A*). *VEGF-A* functions in the development of embryonic structures, during tissue remodelling and for the growth of tumour-induced vasculature. The study of the role of *VEGF-A* during normal development has been significantly complicated by the dominant, haplo-insufficient nature of *VEGF-A*-targeted mutations in mice. We have used morpholino-based targeted gene knock-down technology to generate a zebrafish *VEGF-A* morphant loss of function model. Zebrafish *VEGF-A* morphant embryos develop with an enlarged pericardium and with major blood vessel deficiencies. Morphological assessment at 2 days of development indicates a nearly complete absence of both axial and intersegmental vasculature, with no or reduced numbers of circulating red blood cells. Molecular analysis using the endothelial markers *fli-1* and *flk-1* at 1 day of development demonstrates a fundamental distinction between *VEGF-A* requirements for axial and intersegmental vascular structure specification. *VEGF-A* is not required for the initial establishment of axial vasculature patterning, whereas all development of intersegmental vasculature is dependent on *VEGF-A* signalling. The zebrafish thus serves as a quality model for the study of conserved vertebrate angiogenesis processes during embryonic development.


**Yeast**
*Yeast* 2000; **17**: 294–301.

Research Article

### Distinct requirements for zebrafish angiogenesis revealed by a *VEGF-A* morphant

Aidas Nasevicius[1,2], Jon Larson[1] and Stephen C. Ekker[1]*

[1] *Arnold and Mabel Beckman Center for Transposon Research, Department of Genetics, Cell Biology and Development, University of Minnesota, 6-160 Jackson Hall, 321 Church Street SE, Minneapolis, MN 55455, USA*

[2] *Biochemistry, Molecular Biology and Biophysics Graduate Program, University of Minnesota, 6-160 Jackson Hall, 321 Church Street SE, Minneapolis, MN 55455, USA*

**Correspondence to:* *S. C. Ekker, Arnold and Mabel Beckman Center for Transposon Research, Department of Genetics, Cell Biology and Development, University of Minnesota, 6-160 Jackson Hall, 321 Church Street SE, Minneapolis, MN 55455, USA.* *E-mail: ekker001@mail.med.umn.edu*

Received: 12 September 2000
Accepted: 12 October 2000

### Abstract

**Angiogenesis is a fundamental vertebrate developmental process that requires signalling by the secreted protein vascular endothelial growth factor-A (*VEGF-A*). *VEGF-A* functions in the development of embryonic structures, during tissue remodelling and for the growth of tumour-induced vasculature. The study of the role of *VEGF-A* during normal development has been significantly complicated by the dominant, haplo-insufficient nature of *VEGF-A*-targeted mutations in mice. We have used morpholino-based targeted gene knock-down technology to generate a zebrafish *VEGF-A* morphant loss of function model. Zebrafish *VEGF-A* morphant embryos develop with an enlarged pericardium and with major blood vessel deficiencies. Morphological assessment at 2 days of development indicates a nearly complete absence of both axial and intersegmental vasculature, with no or reduced numbers of circulating red blood cells. Molecular analysis using the endothelial markers *fli-1* and *flk-1* at 1 day of development demonstrates a fundamental distinction between *VEGF-A* requirements for axial and intersegmental vascular structure specification. *VEGF-A* is not required for the initial establishment of axial vasculature patterning, whereas all development of intersegmental vasculature is dependent on *VEGF-A* signalling. The zebrafish thus serves as a quality model for the study of conserved vertebrate angiogenesis processes during embryonic development. Copyright © 2000 John Wiley & Sons, Ltd.**

**Keywords:** **zebrafish; *VEGF-A*; *fli-1*; *flk-1*; angiogenesis; vasculature; morpholino; morphant**

### Introduction

Signalling by members of the vascular endothelial growth factor (*VEGF*) gene family is implicated in the formation of vasculature during embryogenesis, during wound healing and for the growth of tumour-induced vasculature (for reviews, see Carmeliet and Collen, 1997; Ferrara, 1999). Pioneering work in mice with *VEGF-A* demonstrates the extreme dose responsiveness of the mouse embryo to *VEGF-A* signalling during development. Loss of a single copy of the *VEGF-A* gene induces haplo-insufficient lethality by day 9.5 pc (Ferrara *et al.*, 1996; Carmeliet *et al.*, 1996). This biological hurdle to the genetic investigation of *VEGF-A* requirements during later development has resulted in a series of experiments using conditional knock-out strategies (Gerber *et al.*, 1999; Haigh *et al.*, 2000) or dominant negative proteins (Gerber *et al.*, 1999). A more recent approach to address this problem used intravenous injection of antisense oligonucleotides in pregnant mice to reveal loss of function requirements of *VEGF-A* function during murine embryogenesis (Driver *et al.*, 1999).

Copyright © 2000 John Wiley & Sons, Ltd.

Zebrafish embryos develop externally and have only limited requirements for a functioning circulatory system during early development. For example, embryos with no circulating red blood cells due to porphyria live through the first 3 days of development (Ransom *et al.*, 1996), a time period which includes all of segmentation and organogenesis in the fish embryo. Multiple mutations in cardiovascular development were isolated in the initial large-scale chemical mutagenesis screens (Stainier *et al.*, 1996; Chen *et al.*, 1996; Weinstein *et al.*, 1995). The zebrafish has the potential to rapidly assess the biological role of angiogenic factors required for this essential vertebrate process.

We used morpholino-based oligonucleotides (morpholinos, MO; Summerton, 1999) to create a *VEGF-A* loss of function developmental model to circumvent potential haplo-insufficient genetic complications in zebrafish. These *VEGF-A* morphant embryos display an enlarged pericardium and a modest body size decrease and have severe deficiencies in vascular development. We show that the establishment of the axial and intersegmental vasculature has distinct requirements for *VEGF-A* signalling, as revealed by analysis of the expression of two endothelial markers, the tyrosine kinase *VEGF* receptor, *flk-1* (Sumoy *et al.*, 1997; Fouquet *et al.*, 1997; Thompson *et al.*, 1998) and the early vascular marker, the transcription factor *fli-1* (Thompson *et al.*, 1998; Brown *et al.*, 2000). Initial axial expression of these markers is not altered in *VEGF-A* morphant embryos, while no intersegmental expression of these markers is detected. Both axial and intersegmental vasculature fail to function in *VEGF-A* morphant embryos, however, indicating a role for *VEGF-A* beyond the establishment of *flk-1* and *fli-1* expression in blood vessel formation. Similar phenotypes were observed in some mutations found in chemical mutagenesis screens, suggesting a possible role for these genes in *VEGF-A* signalling in the zebrafish embryo.

### Materials and methods

### Morpholino injections

Morpholino antisense oligonucleotides were purchased from Gene-Tools, LLC (Corvallis, OR). Solutions were prepared and injected as described in Nasevicius and Ekker (2000): VEGF-A-1 (predicted start codon is underlined); 5′-GTATCAA ATAAACAACCAAGTTCAT-3′; VEGF-A-1D4 (four-base mismatch), 5′-GTAaCAAtTAAACA ACCAtGTTgAT-3′: VEGF-A-3, 5′-TAAGAAAG CGAAGCTGCTGGGTATG-3′.

### FITC–Dextran injections

Microangiography was performed similarly to the method described in Weinstein *et al.* (1995). Fluorescein isothiocyanate–Dextran (FITC–Dextran) with a molecular weight of 2 000 000 Da (SIGMA, catalogue #FD-2000S) was used for these studies. The Dextran was solubilized in 1 × Danieau solution (58 mM NaCl, 0.7 mM KCl, 0.4 mM $MgSO_4$, 0.6 mM $Ca(NO_3)_2$, 5 mM HEPES, pH 7.6) at 2 mg/ml concentration. 10 μl of the prepared solution was injected into the sinus venosa/cardinal vein of the anaesthetized 48 h embryo. The visualization and photography was performed on a ZEISS Axioskop 2 microscope using a standard FITC filter set.

### Red blood cell (RBC) fluorescence visualization in *urod* morpholino-injected embryos

The fluorescence of red blood cells was observed and photographed as described in Nasevicius and Ekker (2000).

### RNA localization

Whole mount *in situ* hybridization was performed as described in Jowett (1999). Hybridization was performed at 65°C. Riboprobes for *fli-1* and *flk-1* were synthesized using plasmids zffli-1 and zfflk-1 (Thompson *et al.*, 1998), digested with *Eco*RI and *Sma*I, respectively. T7 polymerase was used for riboprobe synthesis.

### Tissue sectioning and visualization

Embryos with FITC–Dextran visualized vasculature were fixed overnight, embedded into paraffin using standard procedures, and sectioned. FITC–Dextran fluorescence outlining blood vessels in unprocessed tissue sections was visualized using ZEISS Axioskop 2 microscope with a FITC filter set. Histological haematoxylin–eosin staining of the sections was subsequently carried out using a standard protocol.

Copyright © 2000 John Wiley & Sons, Ltd. 

### Digital photography

Bright-field and *in situ* photography was performed on a ZEISS Axioplan 2 microscope using Nikon CoolPix 990 (bright-field) or Kodak DCS 420 (*in situ*) digital cameras. For fluorescent photography, a ZEISS AxioCam or a Nikon CoolPix 990 digital camera was used.

### Results and discussion

Zebrafish *VEGF-A* is expressed during embryogenesis in the anterior nervous system, in mesoderm flanking the prospective heart fields, and in somitic mesoderm that flanks the developing endoderm (Liang *et al.*, 1998). We generated morpholino (Summerton, 1999) antisense oligonucleotides against *VEGF-A* to analyse the requirements of this gene during embryonic development. Morpholinos have been recently shown to be effective at gene inactivation during the first 2 days of zebrafish development (Nasevicius and Ekker, 2000). We term the loss of function effects due to morpholinos a 'morphant' phenotype to distinguish this assessment of gene function from that of classical mutant analyses.

*VEGF-A* morphant embryos develop with no overt phenotype during the first day of development. The *VEGF-A* morphant phenotype at 2 days of embryogenesis, however, consists of an enlarged pericardium, no circulating red blood cells, a slight reduction in neural tube and overall body size, and little or no functioning vasculature (Figure 1B). In a subset of embryos, red blood cell accumulation can be noted in the ventral tail (Figure 1C).

We used two separate fluorescent assays to assess vascular function in detail. First, we generated fluorescent-labelled red blood cells through the inactivation of the uroporphyrinogen decarboxylase (*urod*; Wang *et al.*, 1998) gene using morpholinos (Nasevicius and Ekker, 2000). Red blood cells quantitatively accumulate in the anterior hypochord (Figure 2B). To analyse the vasculature directly, we injected fluorescein isothiocyanate–Dextran (FITC–Dextran) into the sinus venosa/cardinal vein of an anaesthetized 48 h embryo (Figure 2C). This microangiography assay labels the entire vasculature of the zebrafish embryo, including the yolk sac, heart, head, axial and intersegmental blood vessels (Weinstein *et al.*, 1995). These structures are differentially sensitive to *VEGF-A* signalling. At high dose injections of *VEGF-A* morpholino, the only vasculature detectable in these animals using this method is found in the heart and yolk (Figure 2D). The vasculature either fails to form at all or contains no functioning connections to the heart in these embryos. To distinguish between these possibilities we performed histological analyses on these most severely effected embryos (Figure 2G–J). Neither dorsal aorta nor axial vein were noted in the injected embryos (Figure 2I, J). We also observed a frequent but less severe phenotypic class of embryos with heart, yolk and head blood vessels (Figure 2E); no axial or intersegmental vasculature was observed in these embryos, however. The least severe phenotypic classification was represented by embryos with reduced intersegmental vasculature and normal heart, yolk, head and axial blood vessels (Figure 2F). The penetrance of these phenotypic classes is very dose-dependent (Table 1), consistent with the strong dose dependence of *VEGF-A* function in mouse embryos (Ferrara *et al.*, 1996; Carmeliet *et al.*, 1996). Heterozygous mouse *VEGF-A* mutants showed a reduced dorsal aorta detected by histological analysis. Fewer intersegmental blood vessels were also detected by a tissue-specific *lacZ* expression (Carmeliet *et al.*, 1996). Lack of the dorsal aorta was indicated by histological analysis in homozygous mouse *VEGF-A* mutants (Carmeliet *et al.*, 1996), suggesting that the most severe zebrafish morphant classes represent a nearly complete loss of function phenotype. However, while mouse *VEGF-A* mutants also display hearts with underdeveloped myoblasts (Ferrara *et al.*, 1996; Haigh *et al.*, 2000), the heart in zebrafish *VEGF-A* morphants has an essentially normal appearance with a slightly enlarged atrium and ventricle, possibly due to higher cardiac pressure (histological analysis not shown).

We assessed the phenotypic effects of two *VEGF-A* morpholinos of non-overlapping sequence and of a four-base mismatch sequence (see Materials and methods) to confirm the specificity of targeting (Table 2). The four-base mismatch morpholino VEGF-A-D4 demonstrates the sequence-specific nature of the noted effects of the VEGF-A-1 morpholino (Table 2). To test independently for the specificity of targeting to the endogenous *VEGF-A* gene, we used a second morpholino of completely independent sequence (VEGF-A-3). This very potent morpholino caused the same

Copyright © 2000 John Wiley & Sons, Ltd. 

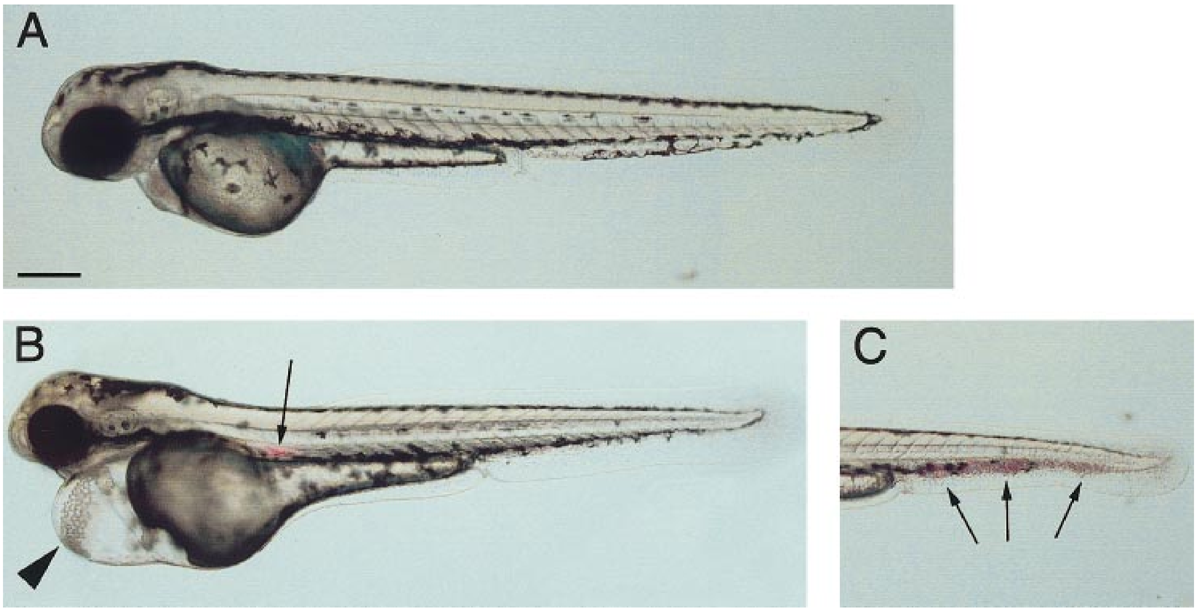

**Figure 1.** Morphological effects of VEGF-A-1 morpholino injection at 36 h. (A) Wild-type embryo. (B, C) Embryos injected with 9 ng VEGF-A-1 morpholino. (B) Loss of vasculature, pericardial oedema (black arrowhead) and blood accumulation in the anterior aorta (black arrow) are prominent (see Table 1 and text). Blood accumulation in tail is also observed (C, black arrows; see Table 1 and text). Bar = 0.2 mm phenotypic effects on development, including a dose-dependent reduction of vascular function (Table 2), pericardial oedema and blood accumulation in the tail (data not shown). The observed differential efficacy might be due to the different secondary structure of the morpholinos or the targeted mRNA region. Alternatively, the effect might be caused by the higher VEGF-A-3 predicted melting temperature due to higher G/C content (48%) as compared to VEGF-A-1 (28%). We conclude that the observed effects are due to morpholino-based inactivation of *VEGF-A* gene function through the specific inhibition of *VEGF-A* transcript translation.

A number of genes whose mutation results in cardiovascular defects were observed in the chemical mutagenesis screens (Stainier *et al.*, 1996; Chen *et al.*, 1996). None of these mutations strongly resemble the *VEGF-A* morphant effects, although mutant embryos with overlapping phenotypes were

Table 1. Microangiography analysis of VEGF-A-1 morpholino injection effects at 48 h

| | **Injected VEGF-A-1 morpholino dose (ng)** | | | | |
|---|---|---|---|---|---|
| **Observed phenotypes (frequency, %)** | **3** | **6** | **9** | **12** | **18** |
| Heart and yolk vasculature only | 3±2 | 3±1 | 21±4 | 38±13 | 39±1 |
| No axial or intersegmental vasculature | 7±4 | 2±1 | 25±3 | 27±13 | 29±9 |
| No/reduced intersegmental vasculature | 67±5 | 78±13 | 51±2 | 36±20 | 30±6 |
| Normal vasculature | 23±10 | 17±16 | 3±1 | 0±0 | 2±3 |
| Pericardial oedema | 15±2 | 35±10 | 49±2 | 55±0 | 57±2 |
| Blood accumulation in anterior hypochord | 0±0 | 1±1 | 5±3 | 3±0 | 8±2 |
| Blood accumulation in tail | 1±1 | 2±2 | 16±8 | 19±0 | 19±7 |
| Total embryo number | 110 | 120 | 105 | 106 | 101 |

Four experiments were performed with each morpholino dose. The phenotype frequencies in every experiment were averaged and entered into the table as average frequency. The differences between the average frequency and the individual experiment frequencies were averaged and entered into the table as a standard error.

Copyright © 2000 John Wiley & Sons, Ltd. 

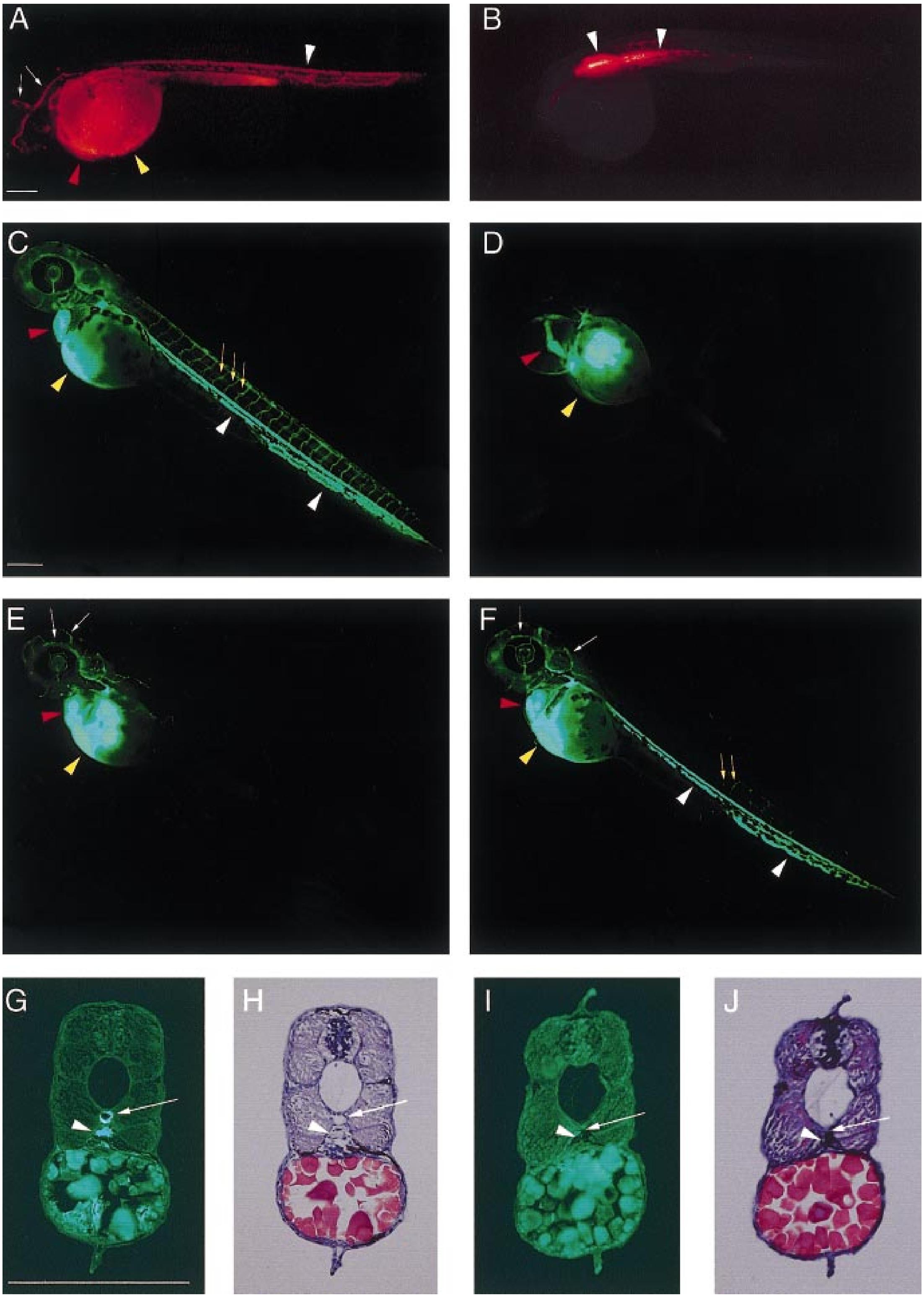

A
B
C
D
E
F
G
H
I
J

Table 2. Microangiography analysis of VEGF-A-D4 and VEGF-A-3 morpholino injected embryos at 48 h

| | **Injected dose (ng)** | | | | |
|---|---|---|---|---|---|
| **VEGF-A-D4 morpholino** | **3** | **6** | **9** | **12** | **18** |
| Heart and yolk vasculature only | 1±1 | 0±0 | 2±3 | 3±0 | 3±0 |
| No axial or intersegmental vasculature | 0±0 | 0±0 | 0±0 | 0±0 | 0±0 |
| No/reduced intersegmental vasculature | 7±0 | 6±3 | 5±2 | 22±1 | 14±3 |
| Normal vasculature | 92±1 | 94±3 | 93±3 | 75±2 | 84±3 |
| Total embryo number | 163 | 116 | 119 | 76 | 73 |
| **VEGF-A-3 morpholino** | **0.5** | **1.5** | **3** | | |
| Heart and yolk vasculature only | 0±0 | 13±13 | 27±11 | | |
| No axial or intersegmental vasculature | 0±0 | 24±24 | 40±13 | | |
| No/reduced intersegmental vasculature | 18±8 | 37±11 | 24±14 | | |
| Normal vasculature | 81±2 | 26±26 | 10±10 | | |
| Total embryo number | 32 | 71 | 69 | | |

Four (VEGF-A-D4 morpholino) or two (VEGF-A-3 morpholino) experiments were performed with each morpholino dose. The phenotype noted. Multiple loci result in embryos with cardiac oedema, and a similar accumulation of blood in the ventral tail fin was noted due to disorganized endothelia in the *scotch tape* (*sco*) mutation (Chen *et al.*, 1996). Several mutations with altered circulation were noted (Stainier *et al.*, 1996; Chen *et al.*, 1996), including *gridlock*, which encodes for a bHLH protein that is required only for arterial development (Weinstein *et al.*, 1995; Stainier *et al.*, 1996; Zhong *et al.*, 2000). The role of these genes in *VEGF* signalling awaits molecular genetic characterization of the remaining loci.

We analysed the expression of two endodermal vascular markers in *VEGF-A* morphant embryos. The transcription factor *fli-1* is a very early marker of vascular cell fate specification (Thompson *et al.*, 1998; Brown *et al.*, 2000). In wild-type 26 h embryos, *fli-1* is expressed in the forming dorsal aorta and axial vein (axial vessels; arrowhead in Figure 3A) and in the intersegmental vasculature in overlying somites (Figure 3A, arrows). In embryos that fail to complete vascular development, only a subset of the vascular expression pattern of these genes is altered, contrary to all vasculature sensitivity to *VEGF-A* knock-down. No detectable intersegmental expression is noted (Figure 3B),

**Figure 2.** Visualization of vasculature defects in VEGF-A-1 morphants using microangiography (Weinstein *et al.*, 1995). (A, C, G, H) Wild-type embryos. (B, D–F, I, J) Embryos injected with 9 ng VEGF-A-1 morpholino. (A, B) 36 h embryos, with red blood cells (RBC) visualized by the injection of 9 ng *urod* morpholino. (A) Fluorescing RBC highlight axial vasculature (white arrowhead), head vasculature (white arrows), yolk sac (yellow arrowhead) and heart (red arrowhead). (B) In the VEGF-A-1 morphant, RBC are localized only to anterior aorta (white arrowheads). (C–F) 48 h embryos with vasculature visualized by FITC–Dextran injection. (C) Wild-type embryo injected with FITC–Dextran. Axial vasculature (white arrowhead), intersegmental blood vessels (yellow arrows), head vasculature, yolk sac (yellow arrowhead) and heart (red arrowhead) are visible. (D) The strongest effect observed upon VEGF-A-1 morpholino injection. The injected FITC–Dextran spreads throughout the yolk sac and heart only. Head, axial and intersegmental blood vessels are not visible. (E) Moderate VEGF-A-1 morpholino injection effect. The injected FITC–Dextran highlights yolk sac, heart and head blood vessels only. (F) Weak VEGF-A-1 morpholino injection effect. No/few intersegmental blood vessels are observed (yellow arrows). Yolk sac, heart, head vasculature and axial blood vessels appear to be normal. In panels A–F, axial vessels are indicated by white arrowhead, intersegmental blood vessels are indicated by yellow arrows, head vasculature is indicated by white arrows, heart is indicated by red arrowhead, and yolk sac is indicated by yellow arrowhead. (G–J) Mid-trunk cross-sections of strongly effected 48 h *VEGF* morphant embryos. (G, I) Fluorescence images of sectioned embryos with FITC–Dextran visualized vasculature. Dorsal aorta (white arrow) and axial vein (white arrowhead) are indicated by FITC–Dextran fluorescence in the wild-type embryo (G), while the vasculature is absent in the injected embryo (I; white arrow and arrowhead indicate where dorsal aorta and axial vein would normally be). (H, J) Haematoxylin–eosin-stained sections shown in (G) and (I), respectively. White arrows indicate present (H) or absent (J) dorsal aorta. White arrowheads indicate present (H) or absent (J) axial vein. Bars = 0.2 mm

Copyright © 2000 John Wiley & Sons, Ltd.

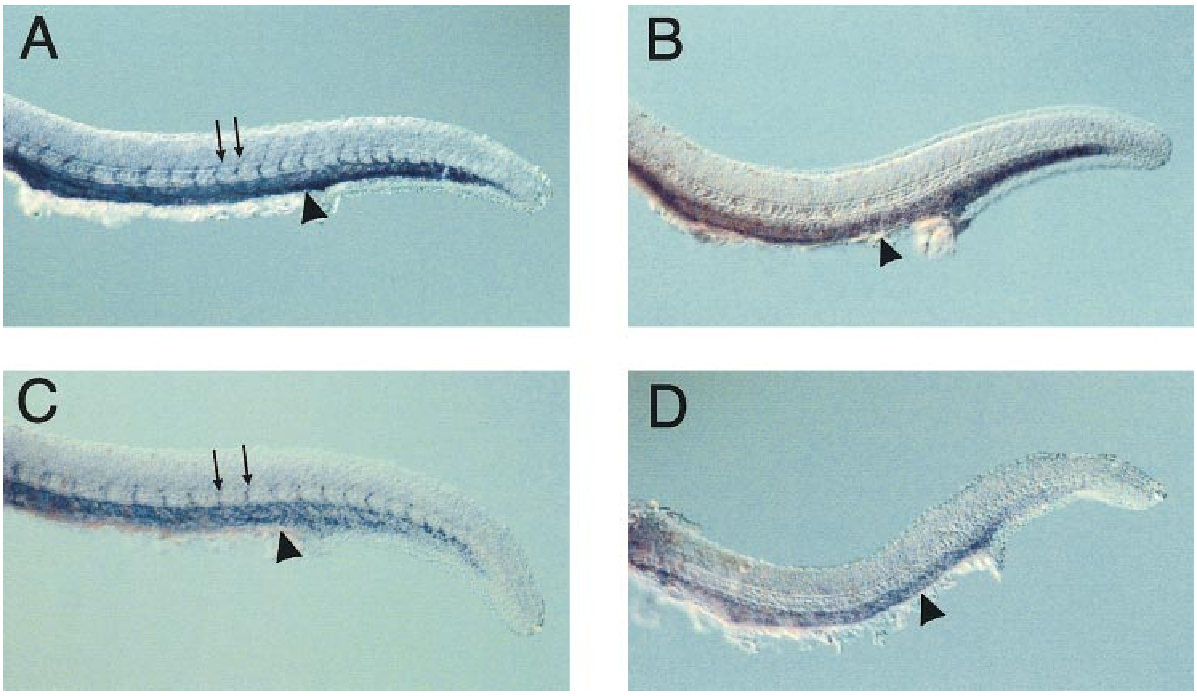

**Figure 3.** Comparative expression of *fli-1* and *flk-1* in 26 h wild-type and VEGF–A-1 morpholino-injected zebrafish embryos visualized by *in situ* hybridization. Anterior is to the left. (A, C) Wild-type. (B, D) Embryos injected with 9 ng VEGF-A-1 morpholino. (A, B) *fli-1* expression. Arrows in (A) depict staining in intersegmental vessels. Arrowheads in (A, B) depict staining in the axial vessels. The VEGF-A-1 morphant in (B) lacks *fli-1* expression in the intersegmental vessels. (C, D) *flk-1* expression. Arrows in (C) depict staining in intersegmental vessels. Arrowheads in (C, D) depict staining in axial vessels. The VEGF-A-1 morphant in (D) lacks *flk-1* expression in the intersegmental vessels coinciding with the exquisite intersegmental vascular endoderm sensitivity to VEGF signalling (Table 1). The cells are either not properly specified or fail to migrate during formation of the intersegmental vessels. Similar results were obtained upon analysis of expression of the *VEGF* receptor, *flk-1*. *flk-1* transcript distribution is very similar to that of *fli-1* in the trunk and tail of wild-type embryos (Figure 3C). In *VEGF-A* morphant embryos, intersegmental but not axial expression is absent (Figure 3D). A significant reduction in *flk-1* gene expression was noted in mouse embryos with no *VEGF-A* activity (Carmeliet *et al.*, 1996). A less extreme lack of *flk-1*-expressing cells in the intersegmental vasculature was also observed in the mouse with the partial and conditional *VEGF-A* knock-out (Haigh *et al.*, 2000). The results in Figure 3 represent work with 9 ng VEGF-A-1 injection-dose embryos; 18 ng injection-dose embryos display the same specific loss of expression only in the intersegmental regions for both *fli-1* and *flk-1* (data not shown).

The distinct responsiveness of the expression of the endothelial marker *fli-1* in intersegmental vessels to *VEGF-A* signalling demonstrates a dual role for *VEGF* during vascular development. First, *VEGF-A* is required for proper axial vessel formation but not for initial axial vessel patterning. Second, *VEGF-A* is required for intersegmental vessel cell specification or migration and, presumably, for subsequent vascular formation. The lack of a requirement for *VEGF* signalling for *flk-1* expression is consistent with previous observations of paracrine modes of *VEGF* signalling (reviewed in Ferrara, 1999). The expression of the *VEGF* receptor *flk-1* is, however, *VEGF*-dependent during intersegmental vascularization. This latter observation suggests a possible autoregulatory loop, functioning during vasculogenesis of the intersegmental vessels.

The strong conservation of *VEGF* function from fish to mammals implicates this as a fundamental vertebrate biological pathway. The use of morpholino-based gene targeting represents a new tool in the genetic repertoire of vertebrate biologists and,

Copyright © 2000 John Wiley & Sons, Ltd. 

combined with the excellent embryology of the zebrafish, will be extremely powerful in the elaboration of gene function for similarly conserved developmental processes. This method will help further elaborate the role of *VEGF* during embryogenesis through the targeted knockdown of other players in this signalling pathway in this outstanding model system.

### Acknowledgements

We thank M. Hammerschmidt and L. Zon for *fli-1* and *flk-1* plasmids and Damien Bates for the suggestion of fluorescent-labelled Dextran for vasculature visualization. This work was supported by the National Institutes of Health, Grant No. GM55877, to SCE.

### References

Brown LA, Rodaway AR, Schilling TF, Jowett T, Ingham PW, Patient RK, Sharrocks AD. 2000. Insights into early vasculogenesis revealed by expression of the ETS-domain transcription factor *Fli-1* in wild-type and mutant zebrafish embryos. *Mech Dev* **90**: 237–252.

Carmeliet P, Collen D. 1997. Molecular analysis of blood vessel formation and disease. *Am J Physiol* **273**: H2091–2104.

Carmeliet P, Ferreira V, Breier G, *et al.* 1996. Abnormal blood vessel development and lethality in embryos lacking a single *VEGF* allele. *Nature* **380**: 435–439.

Chen JN, Haffter P, Odenthal J, *et al.* 1996. Mutations affecting the cardiovascular system and other internal organs in zebrafish. *Development* **123**: 293–302.

Driver SE, Robinson GS, Flanagan J, *et al.* 1999. Oligonucleotide-based inhibition of embryonic gene expression. *Nature Biotechnol* **17**: 1184–1187.

Ferrara N. 1999. Molecular and biological properties of vascular endothelial growth factor. *J Mol Med* **77**: 527–543.

Ferrara N, Carver-Moore K, Chen H, *et al.* 1996. Heterozygous embryonic lethality induced by targeted inactivation of the *VEGF* gene. *Nature* **380**: 439–442.

Fouquet B, Weinstein BM, Serluca FC, Fishman MC. 1997. Vessel patterning in the embryo of the zebrafish: guidance by notochord. *Dev Biol* **183**: 37–48.

Gerber HP, Hillan KJ, Ryan AM, *et al.* 1999. *VEGF* is required for growth and survival in neonatal mice. *Development* **126**: 1149–1159.

Haigh JJ, Gerber HP, Ferrara N, Wagner EF. 2000. Conditional inactivation of *VEGF-A* in areas of collagen 2a1 expression results in embryonic lethality in the heterozygous state. *Development* **127**: 1445–1453.

Jowett T. 1999. Analysis of protein and gene expression. *Methods Cell Biol* **59**: 63–85.

Liang D, Xu X, Chin AJ, *et al.* 1998. Cloning and characterization of vascular endothelial growth factor (*VEGF*) from zebrafish, *Danio rerio*. *Biochim Biophys Acta* **1397**: 14–20.

Nasevicius A, Ekker SC. 2000. Effective targeted gene knockdown in zebrafish. *Nature Genetics* **26** (in press).

Ransom DG, Haffter P, Odenthal J, *et al.* 1996. Characterization of zebrafish mutants with defects in embryonic hematopoiesis. *Development* **123**: 311–319.

Stainier DY, Fouquet B , Chen JN, *et al.* 1996. Mutations affecting the formation and function of the cardiovascular system in the zebrafish embryo. *Development* **123**: 285–292.

Summerton J. 1999. Morpholino antisense oligomers: the case for an RNase H-independent structural type. *Biochim Biophys Acta* **1489**: 141–158.

Sumoy L, Keasey JB, Dittman TD, Kimelman D. 1997. A role for notochord in axial vascular development revealed by analysis of phenotype and the expression of VEGR-2 in zebrafish flh and ntl mutant embryos. *Mech Dev* **63**: 15–27.

Thompson MA, Ransom DG, Pratt SJ, *et al.* 1998. The cloche and spadetail genes differentially affect hematopoiesis and vasculogenesis. *Dev Biol* **197**: 248–269.

Wang H, Long Q, Marty SD, Sassa S, Lin S. 1998. A zebrafish model for hepatoerythropoietic porphyria [see comments]. *Nature Genet* **20**: 239–243.

Weinstein BM, Stemple DL, Driever W, Fishman MC. 1995. Gridlock, a localized heritable vascular patterning defect in the zebrafish. *Nature Med* **1**: 1143–1147.

Zhong TP, Rosenberg M, Mohideen MA, Weinstein B, Fishman MC. 2000. gridlock, an HLH gene required for assembly of the aorta in zebrafish. *Science* **287**: 1820–1824.

Copyright © 2000 John Wiley & Sons, Ltd. 
